# Development of a modified method of handmade cloning in dromedary camel

**DOI:** 10.1371/journal.pone.0213737

**Published:** 2019-04-17

**Authors:** Fariba Moulavi, Sayyed Morteza Hosseini

**Affiliations:** Department of Embryology, Camel Advanced Reproductive Technologies Centre, Government of Dubai, Dubai, United Arab Emirates; University of Florida, UNITED STATES

## Abstract

In this study, a modified method of handmade cloning (m-HMC), which had been originally developed in sheep, was used for somatic cell nuclear transfer (SCNT) in the dromedary camel. The unique feature of m-HMC over current SCNT methods lies in the use of a simple device (a finely drawn micropipette made of Pasteur pipette) for chemically-assisted enucleation of oocytes under a stereomicroscope with improved efficiency and ease of operation. Using this system, the throughput of cloned embryo reconstitution was increased over 2-fold compared to the control SCNT method (c-NT). Stepwise measurement of reactive oxygen species (ROS) revealed that method, steps, and duration of SCNT all influenced oxidative activity of oocytes, but their impact were not similar. Specifically, UV-assisted oocyte enucleation was identified as the major source of ROS production, which explained significantly higher total ROS of reconstituted embryos in c-NT compared to m-HMC. Fusion efficiency (95.3±3.3 vs. 75.4±7.6%) and total efficiency of blastocyst development (22.5±3.0 vs. 14.1±4.3%) were significantly higher in m-HMC compared to c-NT, respectively, and blastocysts of transferable quality were obtained in similar rates (41.9±8.2 vs. 48.0±15.2%, respectively). Significance differences were observed in total cell number (155.3±13.6 vs. 123.6±19.5) and trophectoderm (145±9.5 vs. 114.3±15.2), but not inner cell mass (10.3±4.1 vs. 9.3±5.3) counts between blastocysts developed in c-NT compared to m-HMC, respectively. However, expression of key developmental genes (*POU5F1*, *KLF4*, *SOX2*, *MYC*, and *CDX2*) was comparable between blastocysts of both groups. The introduced m-HMC method might be a viable approach for efficient production of dromedary camel clones for research and commercial utilization.

## Introduction

Animal cloning by somatic cell nuclear transfer (SCNT) has been the subject of much attention in recent years and as a result, several mammalian species have been cloned [1]. Somatic cell cloning in camelids, however, has proven an especially inefficient technology due to the biological and technical problems and the birth of cloned camels has still only been reported from one laboratory [2]. High genetic merit dromedary camels (*Camelus dromedarius*) with exceptional capacities in racing and in production of milk and meat are very valuable genetic resources to regenerate particular populations of camels [3]. Hence, it is expected that the demand for large scale production of cloned dromedary offspring for both research and commercial utilizations will increase. To meet this increasing demand, the efficiency and throughput of camel somatic cell cloning should be improved. The SCNT method used in camels is the original method introduced by Willadsen [4] in spite of its known disadvantages including expensive equipments, high manipulation skills, and low throughput and efficiency [5–8].

The potential application of a handmade method of SCNT that is improved in both efficiency and throughput is considerable. The current method of handmade cloning (HMC), developed by Vajta et al. [9], is based on blind bisection of MII-oocytes using an ultra-sharp blade, H33342 staining and selection of half cytoplasts free of MII-chromosomes under ultra violet (UV) light of an inverted microscope, and fusion of a somatic cell with two half cytoplasts to restore the original oocyte volume. This method, however, wastes 50% of the oocyte starting material during enucleation which is a major disadvantage, especially when oocyte availability is a limiting factor [10]. UV-radiation has established adverse effects on membrane integrity [11], protein synthesis, mitochondrial copy number, viability [12] and development [13] of oocytes. In addition, fusion of two cytoplasts potentially increases the incidence of mitochondrial heteroplasmy [10]. Another potential disadvantage of HMC is that large deformation applied to oocytes during bisection and fusion of two cytoplasts induces a high mechanical stress with subsequent adverse effects on gene expression, actin polymerization and even cell viability [14, 15].

We have developed an alternative approach of zona-free and micromanipulation-free (handmade) cloning method that circumvents these problems of SCNT and further improves throughput, ease of operation and reproducibility in comparison to the current methods [16, 17]. Major elements of this modified handmade cloning (m-HMC) comprise: (1) demecolcine-treatment of zona-free metaphase-II (MII) oocytes to induce cytoplasmic protrusion of MII-chromosomes, (2) oocyte enucleation by removal of the cytoplasmic protrusion of MII-chromosomes with the help of a finely drawn glass Pasteur micropipette (inner diameter of tip ± 10–15 μm) controlled by hand under a stereomicroscope (without UV-irradiation), (3) adherence of donor cell to cytoplast using phytohemagglutinin (PHA) for bulk electrofusion, and (4) group culture of reconstructed oocytes in wells to avoid aggregation of embryos. The described technique showed certain efficiency for large scale production of cloned blastocysts and offspring in sheep [18] and goat [19].

The present study describes the first application of the m-HMC technique in dromedary camel. The objective of our research concentrated on the establishment of m-HMC for large scale production of cloned dromedary embryos in commercial agriculture.

## Material and methods

Unless otherwise specified, all chemicals and media were obtained from Sigma Chemical Co. (St. Louis, MO, USA) and Gibco (Grand Island, NY, USA), respectively. The full experimental procedures were performed in accordance with the government of United Arab Emirates' animal care and use guidelines.

### Oocyte preparation and in vitro maturation (IVM)

The procedure of dromedary camel IVM was as described previously [20]). Briefly, camel ovaries collected from a slaughterhouse and stored at 10°C in normal saline for 24–48 h before being used for the aspiration of superficial follicles (2–8 mm) using an 18-gauge needle into HEPES-buffered tissue culture medium 199 (HTCM199) supplemented with 3 mg/ml polyvinylpyrrolidone (PVP) and 2 IU/ml heparin. Cumulus oocyte complexes (COCs) with homogeneous cytoplasm and at least three compact layers of cumulus cells were selected for culture in groups of 30–35 in 500 μl of IVM medium prepared in Nunc 4-well dish at 38.5°C, 6% CO_2_ and maximum humidity for 30–32 h. IVM medium was comprised of TCM199 supplemented with 10% fetal calf serum (FCS), 2.5 mM Na-pyruvate, 50 μg/ml gentamycin, 1 mg/ml estradiol-17β, 10 μg/ml FSH, 10 μg/ml LH, 1 mM L-glutamine, 0.1 mM cysteamine, and 10 ng/ml EGF. Following IVM, cumulus cells attached to the oocytes were removed by brief vortexing in 0.1% hyaluronidase in HTCM199 + 10% FCS.

### Nuclear donor cells preparation and cell cycle synchronization

Three independent somatic cell lines were used: (1) adult ear skin fibroblasts taken from an adult dromedary elite male camel (age 11 to 12 years) from the private champion racing camel farm, (2) adult ear skin fibroblasts derived from post mortem body of an elite female camel from the reproductive herd of our center, (3) cumulus cell line derived from antral follicles of a slaughtered healthy camel. Skin biopsies were cut into small pieces and cultured in Dulbecco's modified Eagle medium F-12 (DMEM/F-12) containing 10% FCS, 100 U/ml penicillin and 100 μg/ml streptomycin at 37°C, 6% CO_2_ and maximum humidity. The explants were removed after proliferation and establishment of monolayer. Cumulus cells were washed three times by centrifugation and cultured as described for fibroblasts.

For both fibroblast and cumulus cultures, confluent monolayer was trypsinized (0.25% trypsin and 0.05% EDTA for 5 min) and passaged to propagate the cells for cryobanking. The fibroblast lineage was confirmed by morphology assessment and immuno-cytochemical staining against vimentin (fibroblast marker) and pan-cytokeratin (epidermal marker). For cell cycle synchronization at G0/G1, cells were serum starved by culture in medium containing 0.5% FCS for 3 days before being used for nuclear transfer.

### The m-HMC procedure

The full procedure for m-HMC with the illustrative videos can be found in the original papers [18, 19].

#### -Production of handheld enucleation micropipette

Disposable glass Pasteur pipettes (150 or 230 mm long, tip ID ~1 mm) were used for production of the handheld enucleation micropipette as described in details previously. In brief, the narrow portion of the pipette (about 2 cm from the tip) was introduced lengthwise into the flame of a Bunsen burner until it softened. A gentle pressure was applied to bend a 45° angle; the pipette was then removed of the flame and immediately pulled the two ends until the middle portion was drawn to a thickness appropriate for oocyte denudation (ID ~100–150 μm). The resulted narrow portion of the bent pipette (about halfway between the bent angle and the narrow tip bend) was heated again, being careful to soften it by brief approaching beside the flame (not over the flame). When it got soft, it was removed from the flame and quickly pulled. This ended up with one functional micropipette that had a 45° angle and a small piece of glass for disposal. The extra-long hair like part of the micropipette was smoothly broken back clean with the help of the sharp tip of the disposal glass. The size and quality of the produced pipette was evaluated under a stereomicroscope equipped with eyepiece graticule and trimming was repeated, if necessary.

The ideal enucleation micropipette should have completely smooth orifice with an ID of 10–15 μm which is slightly larger than the cytoplasmic protrusion of the demecolcine-treated oocytes. The prepared micropipette is connected to a mouth piece by normal tubing system used for making Pasteur mouth pipette.

#### - Zona removal and demecolcine treatment of oocytes

The basic medium used for oocyte manipulation outside incubator was Ca- and Mg-free HEPES-buffered synthetic oviductal fluid (HSOF) supplemented with 1 mg/mL cold soluble PVA (M_r_: 10–30000). Zona pellucida was removed from oocytes with a first polar body by brief incubation in 5 mg/mL pronase in HSOF + 10% FCS on the warm (38.5°C) stage of a stereomicroscope. As soon the zona was dissolved, oocytes were transferred with the minimum amount of media into washing dish containing 200 μL droplets of HSOF + 20% FCS to inactivate the enzyme. Zona-free oocytes were transferred into droplets of HSOF + 10% FCS and further supplemented with 0.4 μg/ml demecolcine. After incubation for 1 h, oocytes with obvious cytoplasmic protrusion were selected and individually transferred into the enucleation droplets (10 μL of HSOF + 0.4 μM demecolcine) prepared in the lid of a 6-cm Petri-dish (Greiner Bio One) under mineral oil.

To investigate whether demecolcine would interfere with artificial oocyte activation and subsequent development, demecolcine-treated and control oocytes were either activated (5 μM ionomycin for 5 min followed by 2 mM 6-DMAP for 4 h) or left non-activated. After culture in mSOF for 48 h, activated and non-activated oocytes were stained with fluorochrome Hoechst 33342 for scoring of pseudo pronuclei formation by an inverted microscope (Olympus IX71, Japan) equipped with a fluorescent system [10].

#### - Oocyte enucleation

**Fig 1:**
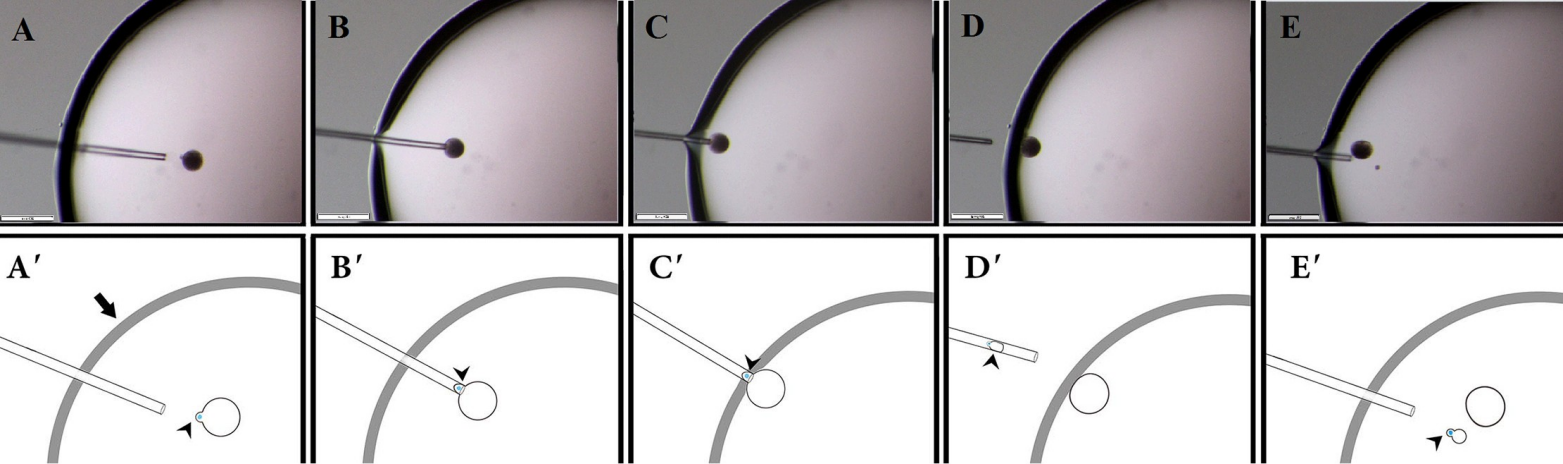
The m-HMC method of oocyte enucleation. Real (A-E) and schematic (A'-E') captions of steps involved in m-HMC method of oocyte enucleation. A, A’: Cytoplasmic protrusion of the oocyte was first positioned at 3 O'clock by moving the micropipette around the oocyte. Arrow indicates the border between enucleation droplet and mineral oil. B, B’: The cytoplasmic protrusion was then allowed to be entered into the micropipette by the capillary action of the micropipette (arrow head) and by a gentle suction by mouth, if needed and allowed it to enter into the pipette. C-D’: While the cytoplasmic protrusion was hold inside the micropipette by gentle mouth suction, enucleation was accomplished by moving the micropipette out from the enucleation droplet into the mineral oil. E, E': As a result, the cytoplasmic protrusion of MII-chromosomes was removed from the oocytes (arrow) and the enucleated oocyte remained in the droplet. The cytoplasmic protrusion remained in the enucleation pipette pushed back into the enucleation droplet (arrow). Bar = 260 μm. Schematic fig was adopted from a previous study[19].

Enucleation was carried out using the handmade enucleation micropipette on a stereomicroscope (Olympus SZX16, Japan) as illustrated in Fig 1. In brief, cytoplasmic protrusion of the oocyte was first positioned at 3 O'clock by moving the micropipette around the oocyte. The cytoplasmic protrusion was then allowed to be entered into the micropipette by the capillary action of the micropipette and by a gentle suction by mouth, if needed. While the cytoplasmic protrusion was hold inside the micropipette by gentle mouth suction, enucleation was accomplished by moving the micropipette out from the enucleation droplet into the mineral oil. As a result, the cytoplasmic protrusion of MII-chromosomes was removed from the oocytes and the enucleated oocyte remained in the droplet.

To verify successful enucleation, removed cytoplasts were stained with 10 mg/ml fluorochrome Hoechst 33342 for 5 min for observation of MII-chromosomes. Oocyte volume changes were calculated from linear measurements of karyoplasts (longitudinally and vertically) under a stereomicroscope equipped with digital camera (Olympus DP26, Japan) and special software in association with internal caliper (Olympus CellSens software, Japan).

#### - Attachment of donor cells

Trypsinized somatic donor cells were resuspended in HSOF199 + 0.5% FCS at a density of 1×10^3^ cells/mL) and aliquoted into 40 μL droplets of HSOF199 covered under mineral oil. About 10–30 individual cells with small size and round bright plasma membrane were selected and added to a droplet of HSOF199 containing 10 mg/mL phytohemagglutinin (PHA). Then, 5–10 cytoplasts were transferred to attachment droplet. Each cytoplast was gently dropped and pressed over a single somatic cell settled to the bottom of the dish with the mouth pipette and incubated for at least 5 min to produce cytoplast-donor cell couplets. Groups of 5–10 couplets were transferred into HSOF/PVA wash drops without PHA to avoid aggregation of couplets.

#### -Electrofusion, artificial activation, and embryo culture

For electrofusion, 10–15 couplets were equilibrated for 2 min in a hypoosmolar (200–210 mOsm) fusion buffer (0.2 M mannitol, 100 μM MgSO_4_, 50 μM CaCl_2_, 500 μM HEPES, 0.05% BSA) at room temperature before transfer into a fusion chamber (BTX, San Diego, CA, 3.2 mm apart electrodes) connected to an ECM 200 (BTX, San Diego, CA). Couplets were first aligned manually and then automatically by applying an alternating current (AC) field (20 V/cm) before fusion using 2×20 μsec direct current (DC) pulses (1 KV/cm), followed by another 10 sec alternative current. Electrofused couplets were incubated in HSOF/PVA for 30 min to score fusion success. Reconstructed oocytes were transferred into Ca and Mg supplemented HSOF (HSOF^+)^ with 1 mg/mL PVA and 10% FCS and incubated for 1.5 h (total incubation time post fusion = 2 h) before activation as described previously [20]. Briefly, reconstructed oocytes were incubated with 5 μM ionomycin prepared in HSOF^+^ containing 1 mg/mL BSA for 5 min followed by washing and incubation in HSOF^+^ containing 30 mg/mL bovine serum albumin (BSA) and then HSOF^+^ containing 3 mg/mL BSA. Oocytes were then incubated in 2 mM 6-Dimethylamino purine (6-DMAP) for 4 h. Activated reconstructs were washed three times in HSOF^+^ containing 3 mg/mL BSA and cultured in a modified formulation of synthetic oviductal fluid (m-SOF) medium (107.7 mM NaCl, 7.15 mM KCl, 0.3 mM KH_2_PO_4_, 25 mM NaHCO3, 3.32 mM sodium lactate, 0.069 mM kanamycin monosulfate, 0.33 mM pyruvate, 1.71 mM CaCl_2_.2H_2_O, 2% BME-essential amino acids, 1% MEM nonessential amino acids, 1 mM L-glutamine, 8 mg/ml fatty-acid free BSA, and 0.1 mM EDTA) for 3 days before transferring the cleaved embryos into fresh m-SOF without EDTA but with 10% charcoal stripped FCS [17]. Embryo culture was performed in groups of 10 in 20 μl droplets of pre-incubated mSOF under mineral oil at 38.5 ºC, 6% CO_2_, 5% O_2_, 89% N_2_ and maximum humidity for 7 days. Zona-free cloned embryos were cultured in wells to avoid their aggregation. Cleavage and further development to the morula and blastocyst stages were recorded at days 3 and 7 post-activation, respectively.

### Control SCNT

The standard zona-intact method of SCNT was used as control in this study (c-NT). In brief, denuded oocytes were treated with 7.5 μg/mL cytochalasin-B in HSOF^-^ for 15 min before transfer into HSOF^-^/FCS droplet on the microscope stage (Olympus, IX71, Japan). A portion of cytoplasm adjacent to first polar body was removed under UV light using a 25 μm micropipette equipped with Narishige micromanipulators (Olympus, Japan). Donor cells were transferred into the perivitelline spaces of enucleated oocytes with the same micropipette. Couplets were first aligned manually and then automatically by applying an AC field (30 V/cm) before fusion using 2×30 μsec direct current (DC) pulses (1.5 KV/cm), followed by another 10 sec alternative current in a normo-osmolar ((270–290 mOsm) fusion buffer. Activation and embryo culture were then performed similar to procedures described to zona-free oocytes, with the exception that embryo culture droplets were without wells.

### Oxidative activity

Since SCNT is a sequential procedure, the steps and the duration of SCNT procedure can affect oxidative activity of oocytes [21]. To understand these, oocytes at different steps of SCNT by either method were used for measurements of reactive oxygen species (ROS) as described elsewhere [21]. In brief, oocytes were incubated in 5 μM working solution of 2,7-dichloro dihydro flourescein diacetate (DCHFDA) in PBS/PVA for 5 min at 39°C and 5% CO_2_. After rinsing in PBS/PVA, oocytes were mounted and examined as described above for measurement of relative fluorescent intensity of DCF (excitation: 450–490 nm; emission: 515–565 nm) and represented as arbitrary units (au). Appropriate positive and negative controls were included. To understand the contribution of SCNT duration on oxidative activity, groups of control MII-oocytes were used for ROS measurements at different intervals post-maturation corresponded to different steps involved in each SCNT method. The oocytes not incubated with DCHFDA were considered as negative control. The oocytes treated with 5% H_2_O_2_ (for 30 min) were considered a positive control group for oxidative stress.

### Assessment of blastocyst diameter and differential staining

The diameter of blastocysts was measured by a stereomicroscope (Olympus SZX16, Japan) equipped with a digital camera (Olympus DP26, Japan) and special software in association with internal caliper (Olympus CellSens software, Japan). Then equal numbers of blastocysts from different size category were pulled together for differential staining. In brief, blastocysts were washed in phosphate buffer saline (PBS), transferred into propidium iodide in Triton (100 μg/mL), immediately transferred into Hoechst 33342 in ethanol (25 μg/mL) and stored at 4°C overnight. After brief washing in PBS, embryos were mounted and examined as described above for counting blue and pink colored nuclei as inner cell mass (ICM) and trophectoderm (TE) cells, respectively [22].

### Quantitative real time PCR (RT-qPCR)

The transcript abundances of 5 key developmental genes (*POU5F1*, *KLF4*, *SOX2*, *MY*C, and *CDX2*) between expanded blastocysts produced by the two SCNT methods. The procedure for RT-qPCR was according to Saadeldin et al. [23] with some modifications in primers (Table 1) and in protocol. In brief, total RNA was extracted using RNeasy Micro kit (Qiagen, Mississauga, ON, Canada) according to the manufacturer’s protocol. The RNA quality and quantity was estimated using spectrophotometer. Reverse transcription program was: 50 cycles of 16°C for 2 min, 42°C for 1 min, and 50°C for 1 s, followed by a final inactivation at 85°C for 5 min. The master mix was prepared using 100 ng of cDNA, 1 mM forward primer, 1 mM reverse primer, and 1 SYBR Green. Three technical replicates of RT-qPCR were conducted for each primer. CT samples of each target gene were normalized to the CT of the reference gene GAPDH, and represented as 2^-ΔΔCT^.

**Table 1:** The qRT-PCR primers. Sequences (5’-3’) of reverse transcription qRT-PCR specific primers of candidate genes expressed in bovine embryos.

Gene	Description	Forward and reverse primers (5’→3’)	Product size (bp)
***POU5F1***	POU class 1 homeobox 1. Critical for somatic cell reprogramming and ESC self-renewal.	**F:** CGAGAGGATTTTGAGGCTGC **R**: GAGTACAGTGTGGTGAAGTGAG	***122***
***SOX2***	SRY (Sex Determining Region Y)-Box 2. Critical for early embryogenesis and for embryonic stem cell pluripotency.	**F:** CTCGCAGACCTACATGAACG **R:** TGGGAGGAAGAGGAAACCAC	***144***
***MYC***	Myc proto-oncogene protein, bHLH transcription factor.	**F:** GGCTAAGTTGGACAATGGCAG **R:** TTCAGCTCGTTCCTCCTCTG	***141***
***KLF4***	Kruppel like factor 4. Critical for somatic cell reprogramming and ESC self-renewal.	**F:** CATCAGCCTCATCCTCGTC **R:** TTCAGCTCGTTCCTCCTCTG	***148***
***CDX2***	Caudal Type Homeobox 2. Trophectoderm differentiation.	**F:** AACCGCAGAGCAAAGGAAAG **R:** AGGGAAGACACAGGACTCAG	***143***
***GAPDH***	Glyceraldehyde-3-Phosphate dehydrogenase. Reference gene.	**F:** GCTGAGTACGTTGTGGAGTC **R:** TCACGCCCATCACAAACATG	***133***

### Statistical analysis

Male fibroblasts were used for comparative experiments between handmade- vs. control-SCNT groups. Data percentages were modeled to the binomial model of parameters by ArcSin transformation. The transformed data were analyzed by one-way ANOVA model of SPSS version 17 (SPSS Science, Chicago, IL, USA). Differences were compared by Tukey's multiple-comparison post hoc test. Data were presented as means ± S.E.M (where ever needed) and differences were considered as significant at *P*<0.05.

## Results

### Demecolcine assisted oocyte enucleation

**Fig 2:**
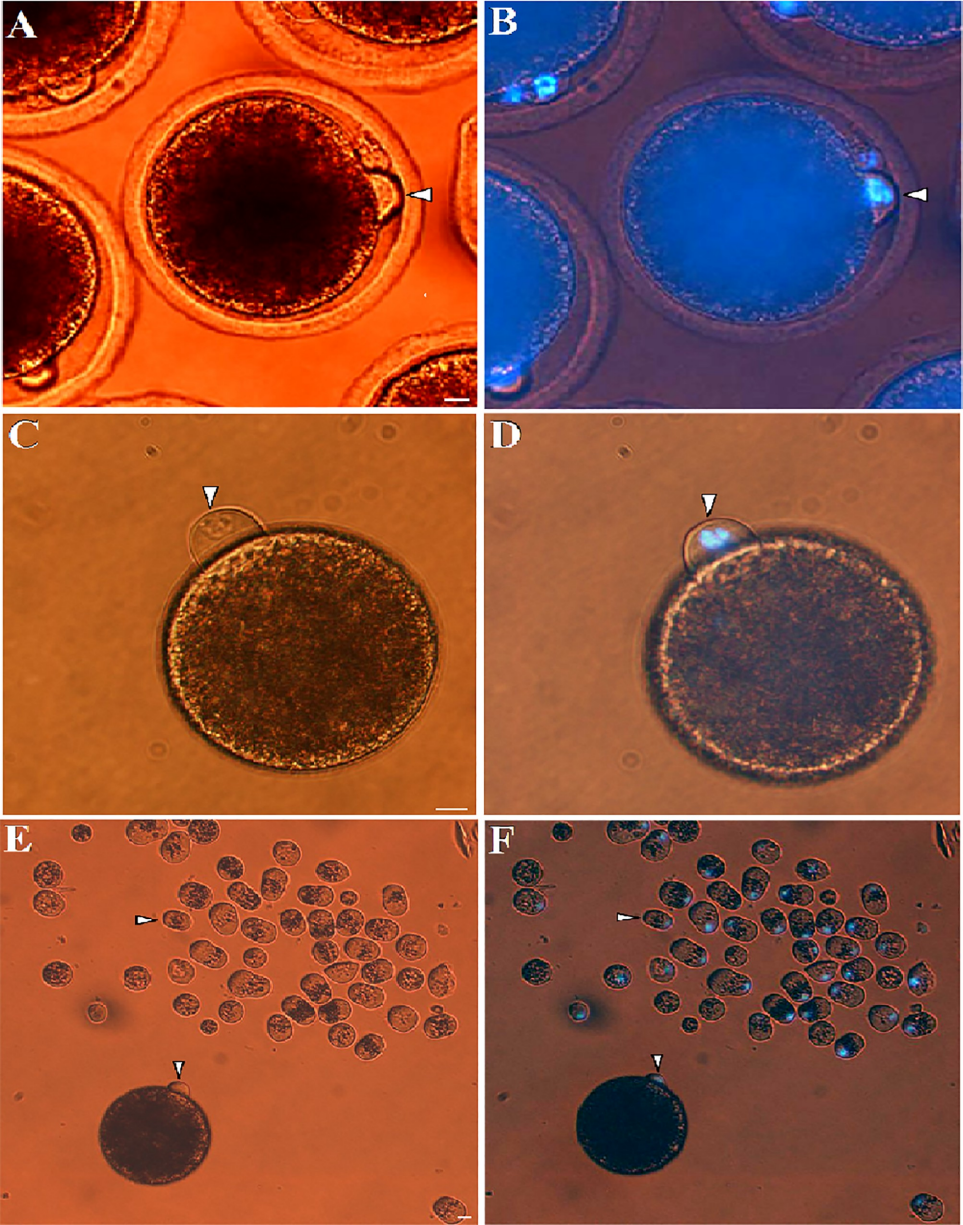
Cytoplasmic protrusion of MII-spindle for m-HMC in dromedary camel. In vitro matured dromedary camel oocytes with characteristic cytoplasmic protrusion (arrow head) observed under bright light (A) and UV-light (B). A typical protrusion (white arrow head) of matured oocytes treated after zona removal and demecolcine treatment observed under bright light (C) and UV-light (D). MII cytoplasmic protrusions (arrow head) removed from MII oocytes using m-HMC method compared with an intact oocyte (asterisk) under bright light (E) and UV-light (F). Bar = 10 μm.

After IVM for 30–32 h, 82.6% of oocytes had a cytoplasmic protrusion beside the first polar body (Fig 2A). Staining with fluorochrome Hoechst-33342 showed that this cytoplasmic protrusion contained entire MII-chromosomes mass (Fig 2B). Demecolcine treatment further increased the incidence rate of cytoplasmic protrusion (>95%). Following zona removal and by dispersal of the first polar body, the cytoplasmic protrusion could be easily detected as a hallmark of MII-chromosomes (Fig 2C and 2D). Demecolcine treatment did not affect spontaneous activation of oocytes (3.3% compared to 5.1% for control). Moreover, artificial activation with ionomycin/6-DMAP resulted in similar pronuclei-formation rates in demecolcine treated- and control- oocytes (86.3% vs. 90.3%, respectively). Demecolcine treatment significantly decreased the rate of oocytes that showed signs of lysis, degeneration or fragmentation during/after handmade enucleation (5.1% compared to 38.5% for non-treated oocytes). According to these results, treatment with demecolcine was applied for handmade enucleation procedure.

### Efficiency and throughput of SCNT

The percentage of oocytes successfully enucleated in m-HMC was comparable to c-NT (91.3±6.0% vs. 95.0±3.3%, respectively) (Table 2). Importantly, the average volume of oocytes reduced in m-HMC was minimal (2.5±1.2%), comparable to c-NT (2.0±1.5%) (Fig 2E and 2F). Short co-incubation of cytoplasts and somatic cells in PHA (10 μg/ml for 1 min) supported efficient production of cytoplast-somatic cell couplets that did not separate during the subsequent manipulation steps. Importantly, cytoplast-somatic cell fusion rates were 95.3% and 75.4% for zona-free and zona-intact couplets, respectively. The difference was significant indicating higher fusion efficiency of m-HMC compared to c-NT group. The overall throughput of m-HMC method in terms of the mean numbers of oocytes enucleated, PHA-agglutinated and electrofused per person per hour were 183, 160, and 229, respectively, which all were significantly higher that the corresponding equivalents of c-NT (93, 69, and 88, respectively). Accordingly, the total throughput of m-HMC was 2.3 fold higher than c-NT method.

**Table 2:** Comparison between efficiency and throughput of m-HMC and c-NT methods.

		Efficiency			Throughput (no./person/h)			
Groups	#	Successful enucleation	Aspirated Oocyte volume	Fusion	Enucleation	Donor cell addition	Fusion	Total throughput
**m-HMC**	195	91.3±6.0% ^a^	2.5±1.2% ^a^	95.3% ^a^	183 ^a^	160 ^a^	229 ^a^	
					*(2*.*0X)*	*(2*.*3X)*	*(2*.*6X)*	*(2*.*3X)*
**c-NT**	155	95.0±3.3% ^a^	2.0±1.5% ^a^	75.4% ^b^	93 ^b^	69 ^b^	88 ^b^	

Male fibroblasts were used for this experiment.

X: Represents increased in throughput of different steps involved in SCNT.

^a,b^: Values with different letters are significantly different (*P < 0*.*05*)

### Oxidative activity of SCNT oocytes

To better understand the SCNT factors affecting oocyte ROS, the level of difference (LD) of ROS were assessed for 3 paired comparisons: (1) m-HMC vs. corresponding non-manipulated (control), (2) c-NT vs. corresponding control oocytes, and (3) m-HMC vs. c-NT. The results presented in Table 3 showed that method of SCNT, the steps involved in SCNT, and also the duration of SCNT, all affected oxidative activity of oocytes. However, their impacts on ROS level of oocytes were not similar. Zona-removal and demecolcine treatment had minor effects on ROS rise in oocytes. However, enucleation, reconstruction, and electrofusion all significantly increased ROS level of oocytes compared to corresponding controls (LD: 1.3, 1.8, and 2.5 arbitrary units (au), respectively). Similarly, ROS levels of oocytes during c-NT manipulations significantly increased compared to control oocytes (LD: 3.2, 3.4, and 4.4 au, respectively). The c-NT procedure significantly increased ROS levels of oocytes during enucleation, reconstitution, and fusion compared to m-HMC (LD: 2.2, 2.0, and 2.4 au, respectively). The mean ROS content of oocytes before SCNT was 2.9 au. Control oocytes in absence of manipulation showed a gradual pattern of ROS increase by time. This time-dependent increase in ROS approached to significant at time points parallel to fusion step in m-HMC (3.5 au) and fusion and reconstitution steps in c-NT (3.7 and 4.0 au, respectively) compared to initial ROS level (2.9 au).

**Table 3:** Differential effects of SCNT method, SCNT steps, and time post maturation on oxidative activity of oocytes.

	DCHFDA fluorescent intensity					
Steps involved	m-HMC vs. control oocytes	LD	c-NT vs. control oocytes	LD	m-HMCT vs. c-NT	LD
**Zona-removal**	3.1 vs. 2.9^a^	0.2	-	-	3.1 vs. (2.9)	0.2
**Demecolcine treatment**	3.2 vs. 2.9^a^	0.2	-	-	3.2 vs. (2.9)	0.3
**Cytochalasin-B treatment**	-	-	2.9 vs. 2.9^a^	0	(3.2) vs. 2.9	0.3
**Enucleation**	4.5 vs. 3.2^a^^b^	1.3*	6.7 vs. 3.5^a^^b^	3.2*	4.5 vs. 6.7	2.2*
**Reconstruction**	5.1 vs. 3.3^a^^b^	1.8*	7.1 vs. 3.7^b^	3.4*	5.1 vs. 7.1	2.0*
**Electrofusion**	6.0 vs. 3.5^b^	2.5*	8.4 vs. 4.0^b^	4.4*	6.0 vs. 8.4	2.4*

Male fibroblasts were used for this experiment.

LD: **L**evel of **D**ifference between the two values compared.

*: Significant difference between ROS levels of the two compared groups (*P < 0*.*05*).

(): values within the parentheses specify steps that do not exist in the related SCNT method. Therefore, values in the bracket show the ROS level of the previous step of the same SCNT method.

^a, b^: Statistical analysis of time dependent changes occur in ROS levels of control MII-oocytes in each SCNT method.Values with different letters are significantly different (*P < 0*.*05*).

### In vitro development of SCNT embryos

**Fig 3:**
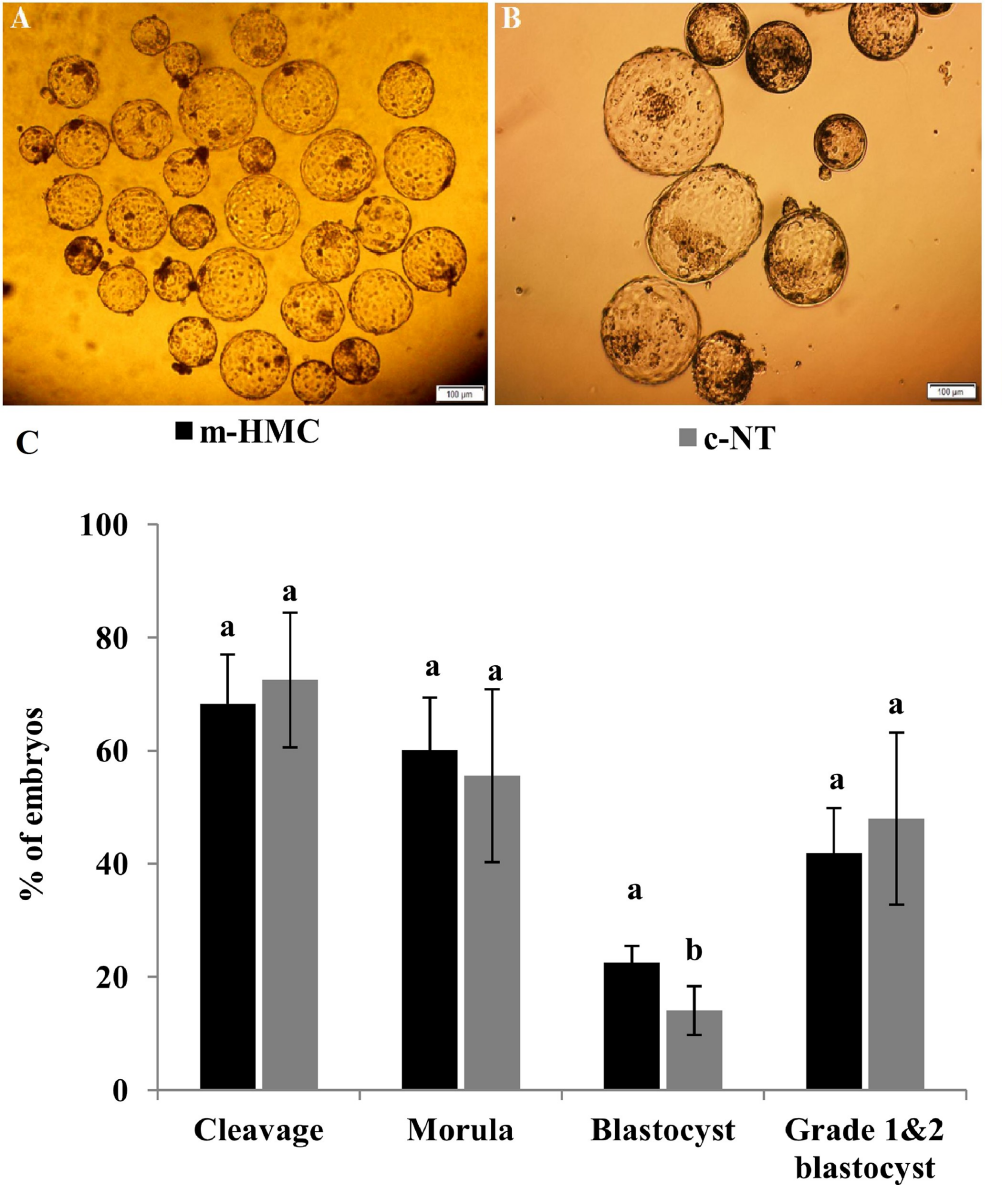
The efficiency of in vitro cloned embryo development by m-HMC in dromedary camel. Day-7 blastocysts produced by m-HMC (A) and c-NT (B) methods. Statistical comparison of in vitro cloned embryo development between m-HMC and c-NT methods. Different letters indicate significant differences (*P<0*.*05*). Bar = 40 μm.

Fig 3A and 3B represent sample pictures of cohort blastocysts produced using m-HMC and c-NT methods, respectively. Fig 3C statistically compares in vitro development of cloned embryos between the two groups. As shown, the cleavage rates were not significantly different between the two methods (68.3±8.7 vs. 72.5±11.9% for m-HMC vs. c-NT, respectively) (Fig 3). The percentages of cleaved embryos progressed to morula were also not significantly different (60.1±9.3 vs. 55.6±15.3%, respectively). However, blastocyst development rate in m-HMC was significantly higher compared to c-NT (22.5±3.0 vs. 14.1±4.3%, respectively). The percentage of grade 1&2 blastocysts was comparable between m-HMC and c-NT groups (41.9 ±8.2 vs. 48.0±15.2%, respectively).

### Blastocyst diameter and differential cell allocation

**Fig 4:**
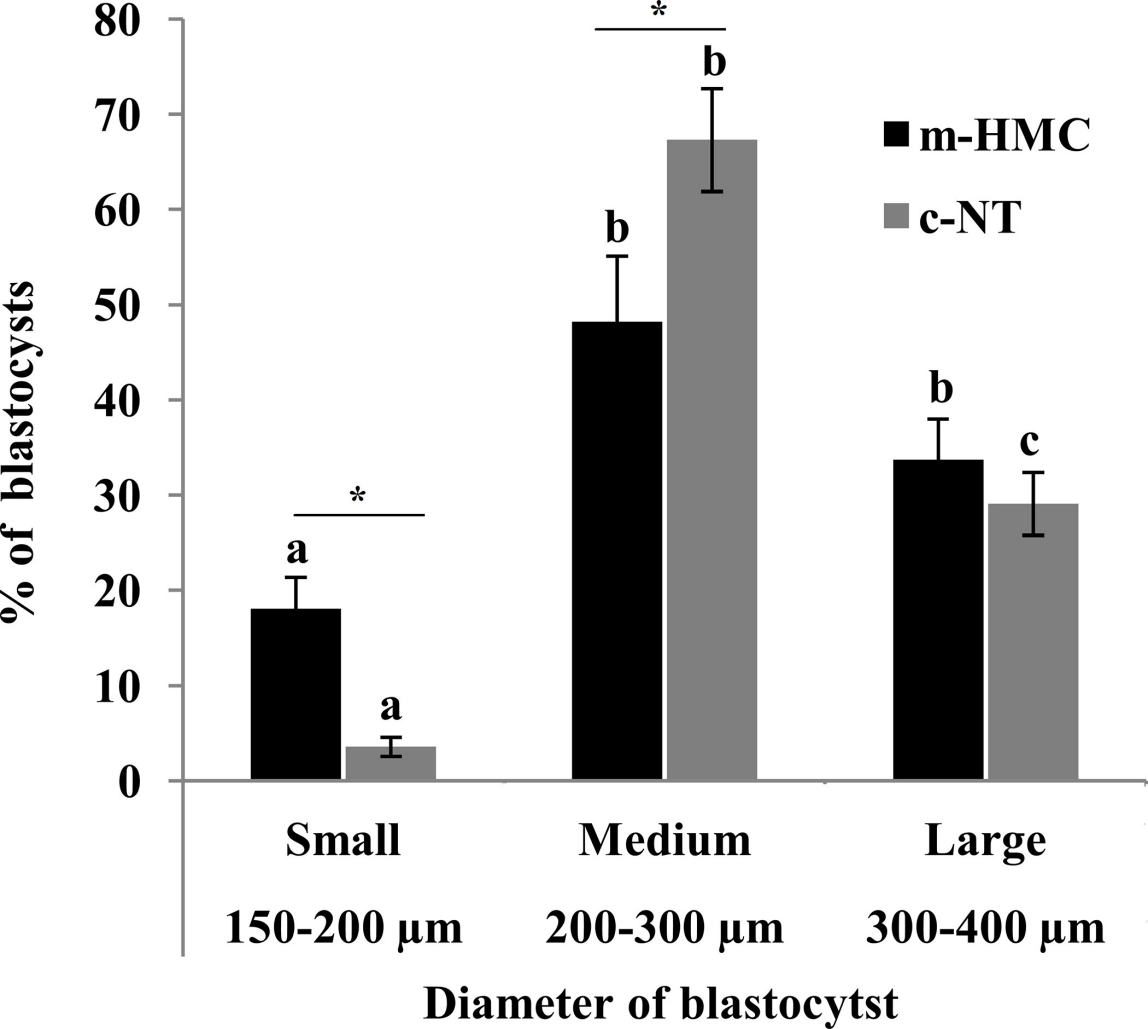
Effect of SCNT method on cloned blastocyst diameter. Statistical comparison of size variance of blastocysts produced by m-HMC and c-NT methods. Different letters indicate significant size differences within the same method (*P<0*.*05*). Asterisk indicates significant differences between the blastocysts of the two SCNT methods with the same diameter (*P<0*.*05*).

**Fig 5:**
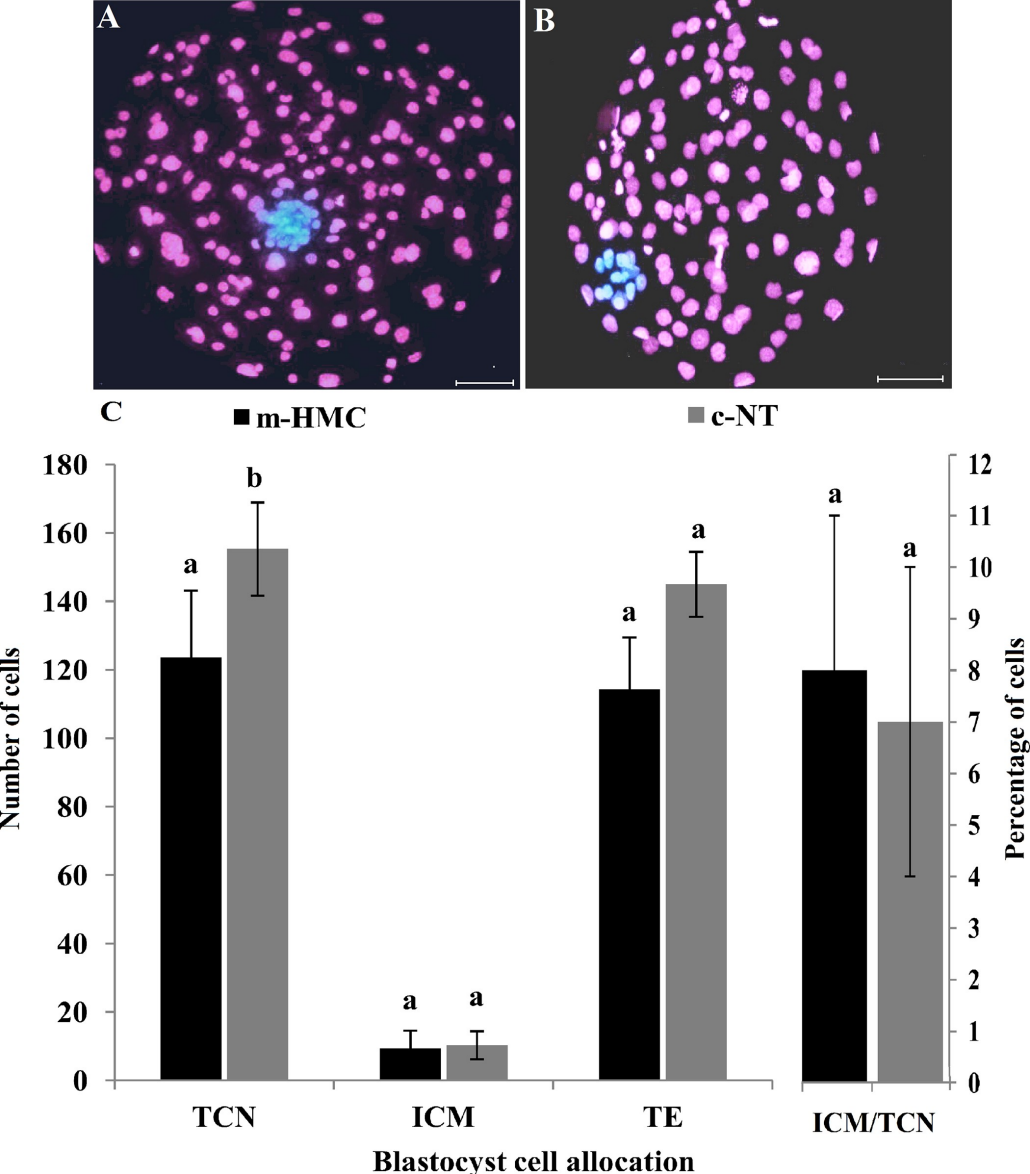
Effect of SCNT method on blastocyst cell number and differential cell allocation. Differential staining images of D-7 blastocysts produced by m-HMC (A) and c-NT (B) methods. Statistical comparison of total cell number (TCN), inner cell mass (ICM) and trophectoderm (TE) counts and ICM/TCN percentages between blastocysts in m-HMC and c-NT. Different letters indicate significant differences (*P<0*.*05*). Bar = 40 μm.

In m-HMC blastocysts, the proportions of small (150–200 μm), medium (200–300 μm) and large (300–400 μm) blastocysts were 18.1, 48.2 and 33.7%, respectively (Fig 4). The proportions of medium and large blastocysts were not significantly different, but both were significantly higher than that of small blastocysts. In c-NT blastocysts, the proportions of small, medium and large blastocysts were 3.6, 67.3 and 29.1%, respectively; all were significantly different from each other. Moreover, the proportions of small and medium blastocysts in m-HMC were significantly lower than the related rates in c-NT. However, there was no significant difference in the proportions of large blastocysts between the two methods. Differential blastocyst staining revealed that the mean total cell number (TCN) of c-NT blastocysts was significantly higher than that of m-HMC blastocysts (155.3±13.6 vs. 123.6±19.5, respectively)(Fig 5A–5C). Trophectoderm (TE) cell number in c-NT also was significantly higher than that of m-HMC (145±9.5 vs. 114.3±15.2, respectively). Nonetheless, inner cell mass (ICM) mean numbers and ICM/TCN ratios were not significantly different between m-HMC and c-NT blastocysts (10.3±4.1 vs. 9.3±5.3 and 0.08±0.3 vs. 0.07±0.3, respectively).

### Gene expression of cloned blastocysts

**Fig 6:**
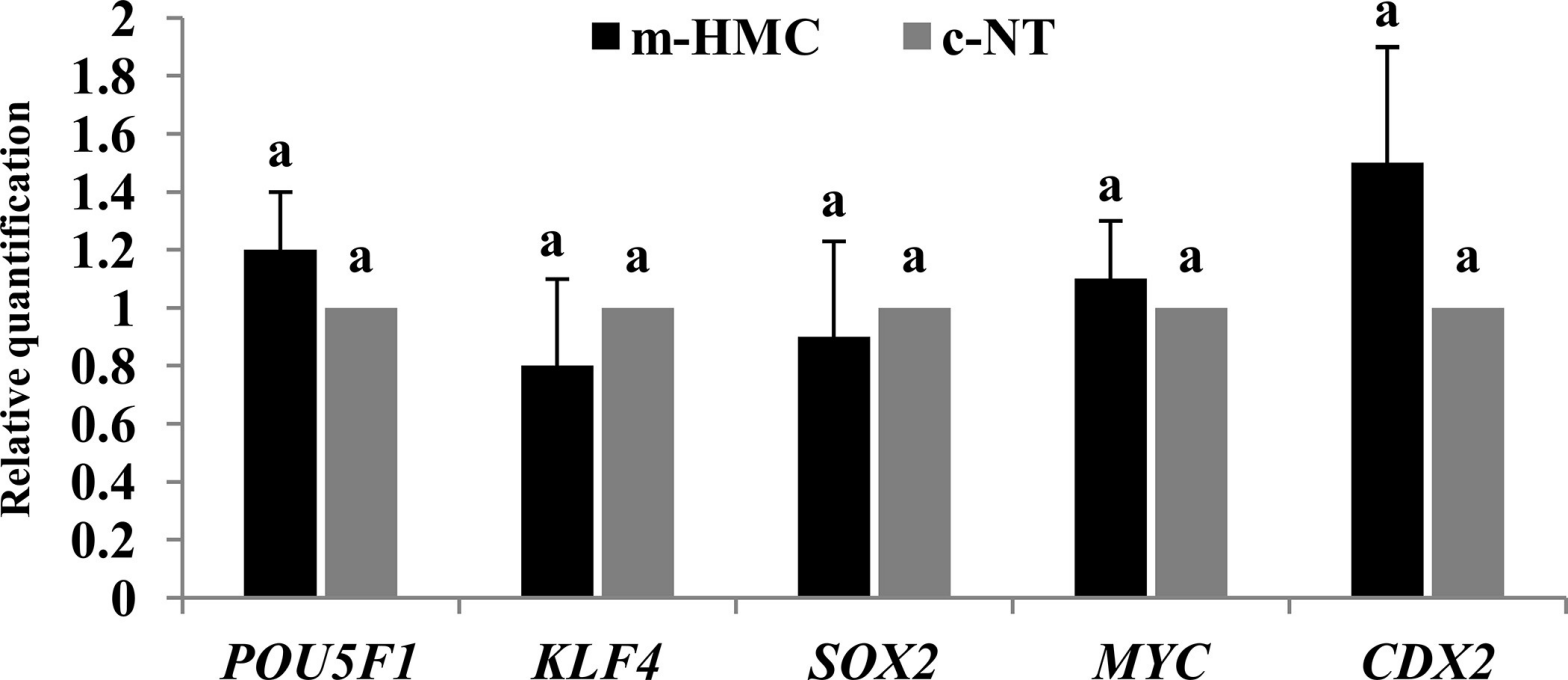
Effect of SCNT method on blastocyst gene expression. Expressions prolife of 5 developmentally important genes (*POU5F1*, *KLF4*, *SOX2*, *MYC*, *and CDX2*) were compared between blastocysts produced by m-HMC and c-NT methods. Different letters indicate significant differences (*P<0*.*05*).

To understand the effect of SCNT method on gene expression of cloned blastocysts, RT-qPCR was performed for 5 developmentally important genes. As shown in Fig 6, the relative expression levels of *POU5F1*, *KLF4*, *SOX2*, *MYC*, and *CDX2* in m-HMC blastocysts compared to c-NT blastocysts showed no significant difference.

### Donor cell type and gender effects on cloned embryo development

**Fig 7:**
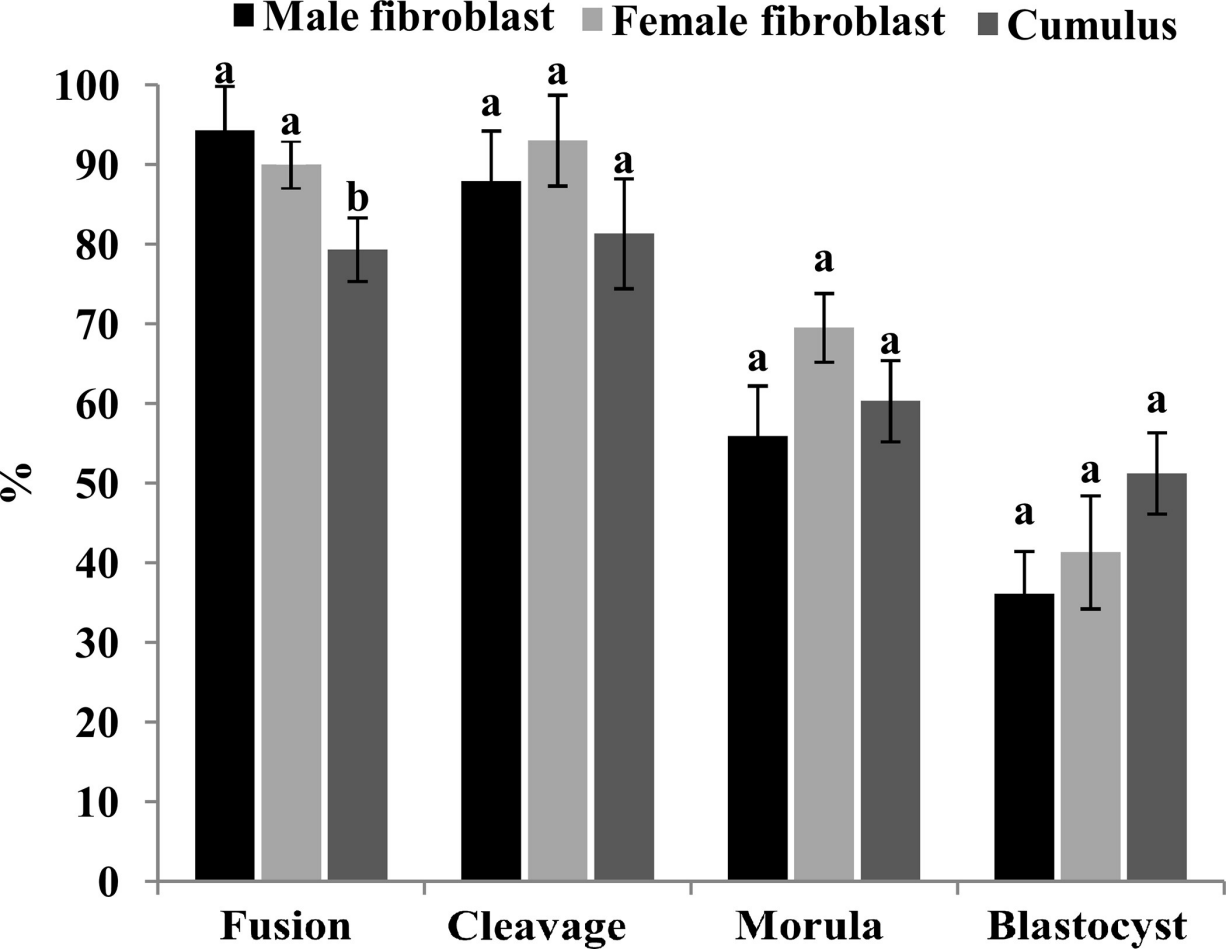
Effect of donor cell type and gender on m-HMC cloned embryo development. Statistical comparison of in vitro development of m-HMC cloned embryos developed using male and female fibroblasts and cumulus cells as nuclear donor cells. Different letters indicate significant differences (*P<0*.*05*).

There was no significant difference in fusion rates of oocytes reconstructed by female and male fibroblasts (94.3±5.5 and 95.0%, respectively), but both rates were significantly higher than fusion rate of cumulus cells (79.3±4.0%) (Fig 7). Beside this, no significant difference was detected in cleavage rates (87.9±6.3, 93.0±5.7, and 81.3±6.9%) and also in the percentages of cleaved oocytes that progressed to the morula (55.9±6.3, 69.5±4.3, and 60.3±5.1%) and blastocyst (36.1±5.3, 41.3±7.1, and 51.2±5.1%) between male and female fibroblasts and cumulus cell lines, respectively.

## Discussion

This study describes the first application of a modified method of handmade cloning (we called it "m-HMC") in dromedary camel. Although the ultimate efficiency in production of clone offspring remains to be evaluated, the established technique significantly increased the throughput of camel cloned blastocyst production in a direct comparison to the standard zona-intact method of cloning (c-NT). In particular, the redox status of reconstituted oocytes and the overall efficiency of cloned blastocyst production were both significantly improved using m-HMC method, although the overall percentage of transferable embryos remained unchanged.

The main advantage of this method over the current zona-intact and zona-free procedures relies in the enucleation step which is performed using an inexpensive handmade enucleation tool under a stereomicroscope without UV according to the below facts: (1) >80% of in vitro matured camel oocytes had a cytoplasmic protrusion of MII-chromosomes which could be easily detected under a stereomicroscope; (2) treatment with demecolcine increased the incidence rate of the cytoplasmic protrusion approximate to 100%; (3) the enucleation tool was a finely drawn Pasteur micropipette that could be reproducibly made in less than 1 min; (4) the efficiency of enucleation, the average reduction in oocyte volume, and the survival of oocytes during/after enucleation were all comparable to c-NT method; (5) the method was twice as fast, reducing average enucleation time to about 15 sec per oocyte.

Although working with zona-free oocytes requires major changes in the manipulation, as demonstrated in details in previous publications [9, 10, 24], it opens up the possibility for improvements in the throughput and efficiency of the other steps of SCNT as well [10]. For example, a short incubation in phytohemagglutinin was enough for efficient and reliable pairing of cytoplasts and somatic cells. A large number of pairs could be positioned simultaneously and fused. Specifically, zona-free pairs required a lower electric field while the fusion rate was significantly increased compared to zona-intact equivalents. These findings are consistent with our previous observations in sheep [18] and goat [19] and also with other studies on zona-free SCNT in bovine [9, 10]. It has been shown that pretreatment of cells with pronase may facilitate fusion by stripping protein from the cell membrane which results in removal of repelling surface charge or changes in membrane fluidity [25–27]. Confirming our observations, Galli et al. [28] reported significantly increased fusion rates with zona-free versus zona-intact cytoplasts in horse. Moreover, the absence of zona further improved automated AC-alignment of a large number of couplets. Culture of reconstructs in microwells prevents aggregation of embryos while allowing them to share a larger common culture media reservoir [5, 24].

Suboptimal culture condition and excess manipulation increase the risk of exposure of gametes and embryos to supraphysiological level of ROS which accounts amongst the most important causes of retarded embryonic development, compromised viability and embryo arrest [29–31]. The ROS production patterns during in vitro maturation and embryo development have been studied in mice [29], sheep [30], bovine [32], and goat [21]. In dromedary camel, intracellular ROS content significantly increased in MI and MII oocytes compared to germinal vesicle oocytes group [33]. We observed that SCNT method, SCNT steps, and manipulation time all affecting ROS content of oocytes, but their relative impacts on ROS rise were not similar. Specifically, enucleation step in c-NT method provoked the greatest rise in ROS content compared to all the other affecting parameters. This may explain that while enucleation, *per se*, is a potential pro-oxidant, exposure of oocytes to UV-radiation is a more significant source of oxidative stress. This along with the increased manipulation time could explain higher ROS content of reconstructed oocytes in c-NT method compared to m-HMC.

The ultimate readout of cloning efficiency in vitro is the yield and quality of blastocyst production. In a direct comparison, m-HMC promoted blastocyst production rate, although blastocysts of transferable quality were obtained at similar rates compared to c-NT. We noticed that m-HMC blastocysts were variable in diameter and this variance was confirmed by variation in cell number and lower mean total cell number of blastocysts compared to c-NT. Differential staining unraveled that this reduction in total cell number was originated from a decrease in TE cells, which are involved in the formation of placenta, while the ICM cells, which are involved in the formation of embryo proper [22], remained unchanged. Variation in embryo diameter is not an in vitro side effect because the same variance has also been reported between in vivo derived embryos in llama, alpaca, and dromedary camel [34–36] and also in horse [37]. Del Campo et al. [34] observed great variability even between single embryos from the same female even in repeated collections and among embryos from different females. For in vivo derived embryos, the variation in embryo has been attributed to variation in ovulation time and thus developmental time [38], superovulatory protocols [39] or intrinsic factors of oocyte quality [34, 35]. Since IVM and IVC procedures and the source of the oocytes were the same for both SCNT methods, the variation in blastocyst diameter and cell number may be explained by the difference between the efficiencies of the two SCNT methods. Because c-NT was less efficient compared to c-HMC, oocyte quality was a key prerequisite, and only oocytes with the superior quality could develop to the blastocyst, while the higher efficiency of m-HMC might allow reconstructed oocytes, even from medium quality, to develop to the blastocyst. Importantly, the expression profile of key developmental genes was comparable between expanded blastocysts of the two SCNT methods. This further confirms this suggestion that blastocyst size variation in m-HMC technique, in comparison to c-NT, may only be related retrospectively to the higher technique efficiency.

As the differentiation and methylation state of different somatic cell types are not the same, the choice of donor cell type has been considered as a determining factor of SCNT outcome [40]. However, there is no consensus regarding the superior somatic cell type for nuclear transfer. Across the three cell lines used for camel handmade-SCNT, the fusion rate of cumulus cells was significantly lower than male and female fibroblasts. We noticed that cumulus cells were smaller in size than fibroblasts. Since somatic AC-alignment and fusion efficiency varies between cell lines and depends on the size ratio between oocyte and donor cell [10, 26], this may explain lower fusion efficiency of cumulus cells. Beside fusion efficiency, donor cell type and gender did not affect the cleavage rates and their development to blastocyst stages, which is consistent with the previous results in sheep [40, 41] and a recent study of in dromedary camel [42]. These results further confirm that this new technique can be used for efficient production of cloned blastocysts from different cell types and gender. In contrast to in vitro development, recent studies suggesting that post-implantation development of cloned embryos can be significantly affected by the donor cell type or gender [42]. Nonetheless, the potential impact of nuclei donor cell lineage on post implantation development of cloned embryos can be easily managed in view of their similar efficiencies for in vitro blastocyst development.

Three previous studies have reported dromedary camel cloning; all used the standard zona-intact SCNT method with in vitro matured [43] and in vivo matured [2, 42] oocytes. They reported no difference in fusion rates of oocytes reconstructed with fetal and adult fibroblasts, granulosa cells and cumulus cells, which are in contrast to the present study, possibly because of different SCNT methods used. The rates of cleavage and blastocysts development using m-HMC in the present study (68.3±8.7 and 22.5±3.0%, respectively) are comparable to the rates obtained by Khatir and Anouassi [43] using in vitro matured oocytes (45–59 and 24–34%, respectively). However, our rates are lower compared to the results obtained by Wani et al. [2] and Wani and Hong [42] using in vivo matured oocytes (88.7 and 51%, respectively). The big gap exists between development of cloned embryos using in vitro (the present study and [43]) and in vivo [10, 42] matured oocytes reflects the critical importance of initial oocyte quality on cloning efficiency and highlights the essential need to optimize IVM in this species. Even though, in agreement with our results, the study of Wani and Hong [42] in camel and our previous study in sheep [40] found no significant effect of donor cell type on cleavage and blastocyst rates of the reconstructed oocytes in camel. This opens the possibility to use fibroblasts as an easy accessible source of nuclei donor cells from both male and female elite camels for conservation efforts and large scale programs of cloning in this valuable species of arid countries.

## Conclusions

Currently, somatic cell cloning in camelids has proven an especially inefficient technique and successful cloning of dromedary camel has still only been reported from one laboratory using in vivo matured oocytes [2]. The search for biological causes underlying low cloning efficiency is confounded by technical aspects of the procedure. Our data suggest that the new version of m-HMC procedure may be a viable alternative to the current available methods for production of cloned blastocyst. Specifically, low costs, simplicity and high throughput and efficiency may be particular advantages this technique in comparison to available methods. Although the ultimate efficiency of this technique has been confirmed by the birth of healthy cloned goat and sheep, our current focus is for a broader application of cloning technology in dromedary camel for both research and commercial utilizations.
